# Expansion of *Betatorquevirus* and/or *Gammatorquevirus* in Patients with Severe Clinical Outcomes of the Liver Diseases

**DOI:** 10.3390/v15081635

**Published:** 2023-07-27

**Authors:** Xiaoan Zhang, William D. Park, Marijn Thijssen, Yanjuan Xu, Long Ping Victor Tse, Mahmoud Reza Pourkarim, Rajeev Aurora, Xiaofeng Fan

**Affiliations:** 1 Division of Gastroenterology & Hepatology, Department of Internal Medicine, Saint Louis University School of Medicine, St. Louis, MO 63104, USA; 2 School of Clinical Medicine, Henan University of Science and Technology, Luoyang 471000, China; 3 Department of Molecular Microbiology & Immunology, Saint Louis University School of Medicine, St. Louis, MO 63104, USA; 4 Laboratory for Clinical and Epidemiological Virology, Rega Institute, Department of Microbiology, Immunology and Transplantation, KU Leuven, 3000 Leuven, Belgium; 5 Saint Louis University Liver Center, Saint Louis University School of Medicine, St. Louis, MO 63104, USA

**Keywords:** anellovirus, virome, next-generation sequencing, hepatitis C virus, hepatocellular carcinoma

## Abstract

Anellovirus (AV) is a ubiquitous virus in the human population. Individuals can be infected with multiple AV genera and species to form a heterogeneous repertoire, termed the anellome. Using advanced methods, we examined the anellomes from 12 paired serum and liver samples, as well as 2701 subjects with different clinical diagnoses. Overall, anellomes are remarkably individualized, with significant among-group differences (Kruskal–Wallis test *p* = 6.6 × 10^−162^ for richness and *p* = 7.48 × 10^−162^ for Shannon entropy). High dissimilarity scores (beta diversity) were observed between patient groups, except for paired serum and liver samples. At the population level, the relative abundance of combinational AV genus *Betatorquevirus* (torque teno mini viruses, TTMV), and *Gammatorquevirus* (torque teno midi viruses, TTMDV) exhibited an exponential distribution with a low bound point at 32%. Defined by this value, the AV TTMV/TTMDV-expanded anellome was significantly enriched among patients with acute liver failure (31.7%) and liver transplantation (40.7%), compared with other patient groups (χ^2^ test: *p* = 4.1 × 10^−8^–3.2 × 10^−3^). Therefore, anellome heterogeneity may be predictive of clinical outcomes in certain diseases, such as liver disease. The consistency of anellome between paired serum and liver samples indicates that a liquid biopsy approach would be suitable for longitudinal studies to clarify the causality of the AV TTMV/TTMDV-expanded anellome in the outcomes of liver disease.

## 1. Introduction

Anellovirus (AV) is a non-enveloped vertebrate virus whose genomes comprise a circular single-stranded DNA. Although AV is ubiquitous among the human population [1], there is currently no firm evidence supporting an etiological role of AV in human disease. Consequently, AV is considered to be a commensal virus and a major component of the human virome [2]. Anellovirus-like sequences are frequently detected in mammals other than humans [3]. These sequences are assigned to the newly created viral family *Anelloviridae* [4]. Phylogenetically, human AVs can be divided into three genera—*Alphatorquevirus* (torque teno virus, TTV), *Betatorquevirus* (torque teno mini viruses, TTMV), and *Gammatorquevirus* (torque teno midi viruses, TTMDV)—based on the AV open reading frame 1 (ORF1). Each genus contains multiple species defined by ≥69% sequence identity in the AV ORF1; currently, there are at least 26 species in TTV, 38 species in TTMV, and 15 species in TTMDV [4]. An individual can be infected with multiple AV genera and species, resulting in a heterogeneous AV population, referred to as the anellome [2]. This raises the question of whether AV plays a role in human health and disease in a similar manner to its bacterial counterpart, the gut microbiota, in which compositional alteration, so-called dysbiosis, is a salient characteristic under numerous human diseases [5]. To this point, we first developed improved methods to measure anellome complexity in a quantitative manner, and subsequently discovered that the relative abundance of AV TTMV and/or TTMDV within an anellome was unusual high in patients with hepatitis C virus (HCV)-associated hepatocellular carcinoma (HCC) [6]. Following this observation, the current study aimed to investigate whether the serum anellome is representative of the liver anellome in terms of AV population structures, and to analyze the statistical relationship between anellome structure and human diseases. We evaluated the anellomes of paired serum and liver samples from 12 patients with chronic HCV infection, and reconstructed the anellome populations of 1646 next-generation sequencing (NGS) data (libraries) from 10 published serum and/or plasma metagenomics studies. Our results show highly individualized anellomes among the study subjects and that anellome complexity can be used as a quantifiable metric in exploring AV’s role in human health and disease.

## 2. Materials and Methods

### 2.1. Patient Samples

Paired serum and liver biopsy tissue samples were obtained from 12 patients with chronic HCV infection (Table 1). These samples were collected for research purposes and archived in the Saint Louis University Liver Center Sample Repository. Written informed consent was obtained from each patient prior to sample collection. No antiviral therapy was given to these patients prior to sample collection. HCV RNA titers were quantitated using Roche Amplicor HCV Monitor (version 2.0) (Roche Diagnostic Systems; Indianapolis, IN, USA). All patients were infected with HCV genotype 1a as determined via line probe assay (Innogenetics, Ghent, Belgium). Liver histology was evaluated using Scheuer’s scoring system [7]. The entire research protocol for the use of these samples was reviewed and approved by the Saint Louis University Institutional Review Board (IRB protocol: SLU10592).

### 2.2. Anellome Sequencing

Anellome sequencing was performed as previously described [6]. In brief, total DNA was extracted from 250 µL of serum using the QIAamp Circulating Nucleic Acid Kit (Qiagen; Valencia, CA, USA), and was eluted into 60 µL of AVE buffer. Frozen biopsy samples of liver tissue, weighing approximately 5 mg, were homogenized using a MagNALyser (Roche Diagnostics; Mannheim, Germany), and DNA extraction was carried out using an AllPrep DNA/RNA Mini Kit (Qiagen). Total DNA was finally eluted into 50 µL of buffer EB and quantified using the Quant-iT PicoGreen dsDNA Assay kit (Qiagen). A 5 µL aliquot of extracted serum DNA (or 200 ng tissue DNA) was used for two-phase rolling circle amplification (2pRCA), which involved a 12 h incubation at 30 °C with two AV-specific primers (1st phase RCA). These two primers were derived from the AV non-coding region and are conserved among human AV species, AV1, 5′-CATT*C*G-3′ and AV2, 5′-CCGA*A*T-3′ (asterisk donates phosphorothioate bond) [6]. The reaction was followed by an additional 6 h at 28 °C with the inclusion of 5′-end-blocked random pentamer primers [6]. The 2pRCA product was purified with the QIAamp DNA mini kit (Qiagen), quantified, and subjected to library preparation with the Nextera XT DNA Sample Preparation kit (Illumina; San Diego, CA, USA). Sequencing was then performed on the Illumina MiSeq (2 × 150-nt paired reads and mid-output) at MOgene (St. Louis, MO, USA).

### 2.3. Determination of Anellovirus Copy Numbers

The AV titers in serum and liver samples were measured using an established real-time PCR protocol targeting the non-coding region (NCR) [6]. This protocol was originally developed in Dr. Maggie’s lab and validated to be comparable with the commercial TTV RGENE kit and digital droplet PCR [8,9,10]. Briefly, a 5 µL aliquot of extracted serum DNA (or 200 ng tissue DNA) was added to a 25 µL reaction volume containing 1× TaqManTM Fast Universal PCR Master Mix, no AmpErase™ UNG (Thermo Fisher Scientific; Waltham, MA, USA), and AV NCR-specific primers AMTS (5′-GTGCCGIAGG TGAGTTTA-3′) and AMTAS (5′-AGCCCGGCCAGTCC-3′) and probe AMTPTU (5′-TCAAGGGGCAATTCGGGCT-3′. The reaction was carried out on the ABI 7500 instrument (Applied Biosystems; Waltham, MA, USA) with initial heating at 95 °C for 10 min, followed by 45 cycles of 95 °C for 15 s and 58 °C for 60 s. AV-negative and AV plasmid-spiked mock serum samples were included as standards. Three technical replicates were conducted on each sample, and the mean value was taken to represent the final AV copy number.

### 2.4. Data Collection from Serum/Plasma Metagenomics Studies

We conducted a literature search of PubMed between 2012 and 2022 using the key words “Torque teno virus” (TTV), “anellovirus”, or “virome”. A study would be included if it used plasma or serum samples for metagenomic sequencing with raw sequencing data available in the National Center for Biotechnology Information (NCBI) Sequence Read Archive (SRA). Sequencing data from these studies were used to elucidate potential anellome heterogeneity among human diseases.

### 2.5. Analysis of Anellome Composition

All data from paired liver/serum samples and published metagenomics studies were subjected to the bioinformatics pipeline described previously [6]. After read quality control, AV-related reads were extracted at three similarity levels: mapping (high level), blastx (moderate level), and profile hidden Markov model (HMM) (low level). AV reads were de novo assembled using SPAdes (version 3.15.2) [11]. Resulting contigs (≥300 nt) were collapsed at 90% similarity in a Cluster Database with High Identity with Tolerance (CD-HIT) [12], then assigned for AV species attributes using blastx against the ORF1s from 79 AV reference sequences [4]. Based on blastx-defined positions in the contigs, the AV ORF1 reads from each dataset were re-extracted and used as input for genome abundance similarity correction (GASiC) to estimate relative genome abundances via read alignment [13]. Abundance data were then summarized at the level of AV species and compared among patient groups. Statistical distribution patterns of the relative abundances of AV genus TTMV and TTMDV were explored at the population level using Clauset’s formula implanted in the R package poweRlaw [14,15]. A low bound point was determined under a defined statistical pattern.

### 2.6. Analysis of Anellome Diversity

The number of AV species in each anellome was counted to determine the richness of the anellome, and the alpha diversity was evaluated using Shannon entropy computed in the R package QSutils [16]. Based on Bray–Curtis or Yue–Clayton formulae [17,18], anellome dissimilarity between patient groups (beta diversity) was conducted using in-house scripts available in the Supplementary Materials. Dissimilarity matrices were then applied to principal coordinate analysis (PCoA) in the R stats package [19].

### 2.7. Statistical Analyses

The Shannon entropy and relative abundances of AV species were compared using the Kruskal–Wallis test for multi-group comparison. Pairwise comparisons were carried out using the Wilcoxon test. All analyses were performed and visualized using the R packages tidyverse, ggpubr, and rstatix [20,21,22]. Categorical data from cross analyses were evaluated for statistical significance using the χ^2^ test with Yate’s correction or Fisher’s exact test. Between-group comparisons (serum vs. liver) were carried out using the two-tailed Student’s *t*-test. Data are expressed as mean ± standard deviation, and *p* < 0.05 was considered statistically significant.

### 2.8. Phylogenetic Analysis

The liver transplantation cohort (PRJNA660895), one of the metagenomics studies targeted through our literature search, consisted of 24 cases including 14 patients with HCC and 10 with cirrhosis. Plasma samples were collected from each patient at six time points, two before and four after surgery, within an average of a 5-year period [23]. We conducted phylogenetic analysis on selected cases of this cohort, with AV ORF1 being detected at multiple time points. Briefly, AV ORF1 amino acid sequences from selected cases and from AV reference species were aligned using the Multiple sequence Alignment Fast Fourier Transform (MAFFT) program (version 7.505) [24], and then trimmed manually. The alignment with 271 amino acid sites was used to construct maximum-likelihood trees in the Molecular Evolutionary Genetics Analysis (MEGA) software package (version 10) with a bootstrap test to estimate the reliability of tree topologies [25].

## 3. Results

### 3.1. Comparison of Serum and Liver Anellomes

**Figure 1:**
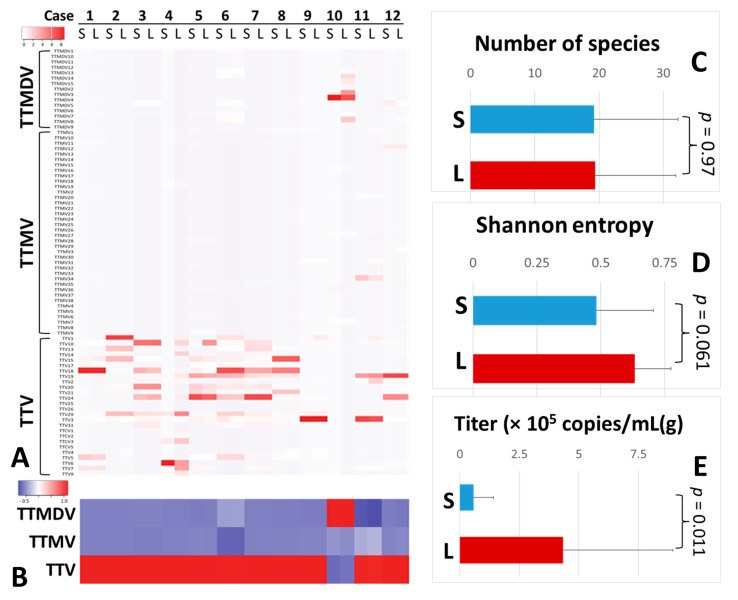
Anellovirus profiles of paired serum and liver tissue samples. Twelve paired serum and liver samples were compared with regard to the anellome composition resolved at the level of AV species (**A**) or genus (**B**). Anellome richness (**C**), Shannon entropy (**D**), and anellovirus titer (**E**) were also compared between serum and liver samples.

**Figure 2:**
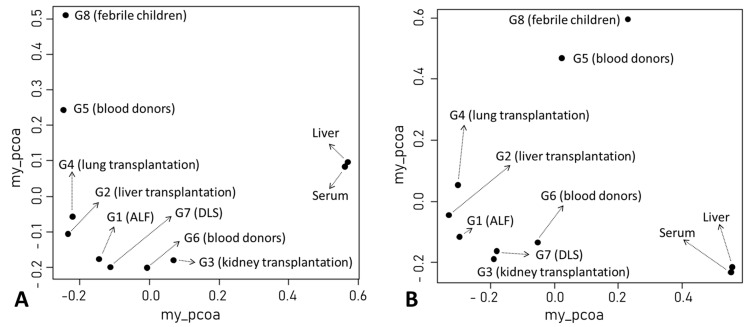
Results of principal coordinate analysis of anellomes among ten groups. The analysis was conducted using either Bray–Curtis (**A**) or Yue–Clayton (**B**) dissimilarity matrices. The clinical diagnosis of each patient group is indicated. DLS, dengue-like symptom.

We found that TTV was dominant in paired serum/liver anellomes from 11 of 12 patients with chronic HCV infection. In the other patient (case 10), the prevailing AV genus in the anellome was the TTMDV (Figure 1A,B). Serum and liver samples were similar in terms of numbers of AV species (19.2 ± 13 vs. 19.4 ± 12.5, *p* = 0.97) (Figure 1C). However, the Shannon entropy (alpha diversity) of the liver anellome was increased with marginal significance compared with the serum anellome (0.63 ± 0.14 vs. 0.48 ± 0.22, *p* = 0.061) (Figure 1D). On average, the AV titer was 7.55 times higher for liver samples than for serum samples (4.33 ± 4.61 × 10^5^ copies/g vs. 0.57 ± 0.82 × 10^5^ copies/mL, *p* = 0.011) (Figure 1E). Furthermore, compared with other patient groups, anellomes from paired serum and liver samples showed the lowest dissimilarity scores (Bray–Curtis score = 0.2 or Yue–Clayton score = 0.1) and almost identical positions in PCoA analysis (Figure S1 and Figure 2).

### 3.2. Anellome Diversity among Patient Groups

**Figure 3:**
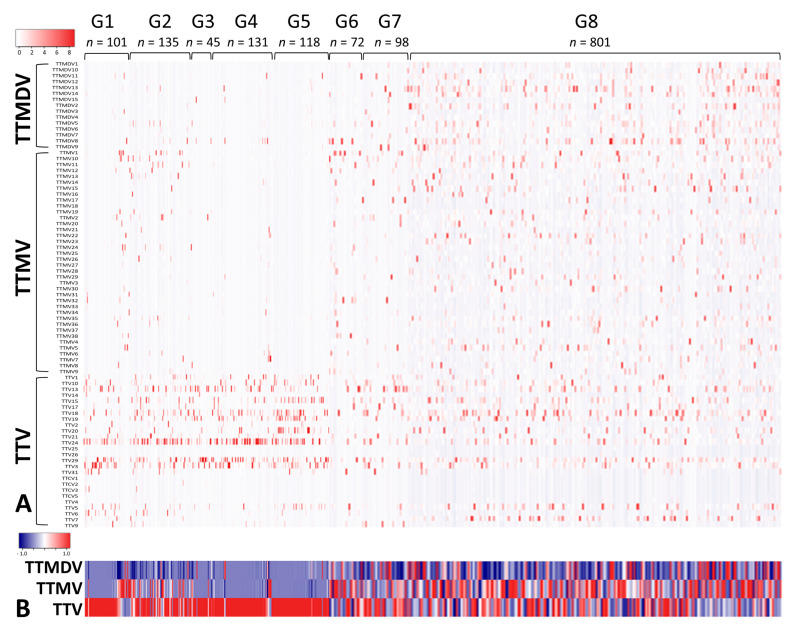
Serum/plasma anellomes of subjects in relation to patient groups. Anellome compositions, with the determination of relative abundance, were summarized at the level of anellovirus species (**A**) and genus (**B**). Groups G1–8 stand for acute liver failure, liver transplantation, kidney transplantation, lung transplantation, blood donors, blood donors from Spain, dengue-like symptoms, and febrile children, respectively.

**Figure 4:**
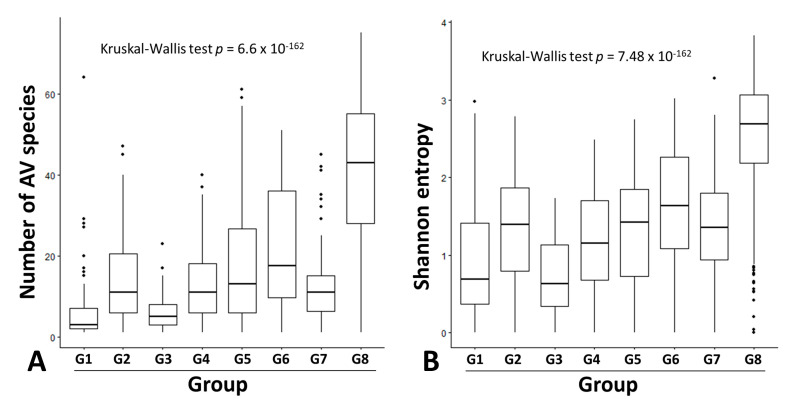
Box and whisker plots representing anellome diversity. The number of anellovirus species (**A**) and Shannon entropy (**B**) among the eight groups are represented by boxes (second quartile, median and third quartile diversity) and whiskers (minimum and maximum diversity). Dots indicate outliers. Among-group comparisons were carried out using the Kruskal–Wallis test. Groups G1–8 stand for acute liver failure, liver transplantation, kidney transplantation, lung transplantation, blood donors, blood donors from Spain, dengue-like symptoms, and febrile children, respectively.

Our literature search identified 10 serum/plasma metagenomics studies with raw sequencing data available in the NCBI Sequence Read Archive. These studies covered diverse clinical situations, including acute liver failure [26], liver transplantation [23], kidney transplantation [27], lung transplantation [28,29], blood donors [30,31,32,33], dengue-like symptom [34], and febrile children (Table 2) [35]. Based on clinical diagnosis and NGS library preparation strategy (pooled vs. non-pooled samples), we classified 10 serum/plasma metagenomics studies into eight groups, G1–8 (Table 2). Of the 1646 NGS data (libraries) from 2701 subjects, 1501 had ≥100 AV reads and simultaneous detection of AV ORF1 contigs with ≥1× coverage. The AV quantitative categorization from 1501 NGS data revealed a diverse pattern of anellome composition across the subjects (Figure 3). Among the 1501 data, 66.6% of assembled contigs could be assigned to all 79 AV reference species with different frequencies, including 4 simian species (*Torque teno chlorocebus virus 1* [TTCV1], TTCV2, TTCV3, and TTCV4) (Figure S2). Overall, TTV20 was the most abundant AV species (average 13.9%) within anellomes from the 1501 NGS data (Figure S2). The number of AV species (richness) and Shannon entropy (alpha diversity) varied significantly between the eight groups (Figure 4) (*p* = 6.6 × 10^−162^ for richness and *p* = 7.48 × 10^−162^ for Shannon entropy from multi-group Kruskal–Wallis test). Accordingly, most pairwise comparisons between the groups revealed statistically significant differences (Table S1). Similarly, high dissimilarities calculated via the Bray–Curtis or Yue–Clayton formulae were observed between the groups, except for the paired liver/serum cohort (Figure S2). Our PCoA analysis revealed that all groups were separated from each other, but the G1, G2, G3, G4, G6, and G7 were well distanced from G5, G8, and the paired liver/serum cohort (Figure 2).

### 3.3. The Prevalence of TTMV/TTMDV-Expanded Anellomes among Patient Groups

**Figure 5:**
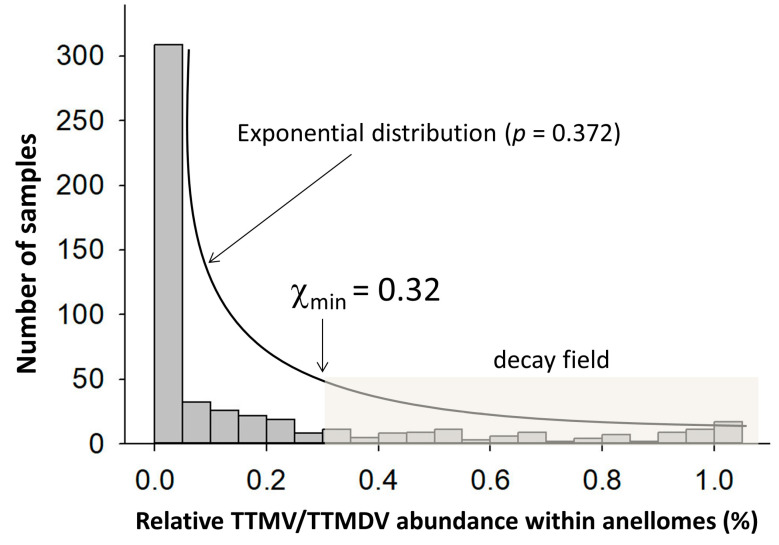
Histogram showing the relative abundance of combinational TTMV and TTMDV in the anellomes from 530 serum/plasma samples. The data distribution fit an exponential distribution, in which most samples (78.7%) had TTMV and TTMDV shrunk within the anellome. The χ_min_ was calculated to be 32, which put 113 samples (21.3%) in the decay region (shaded area).

**Figure 6:**
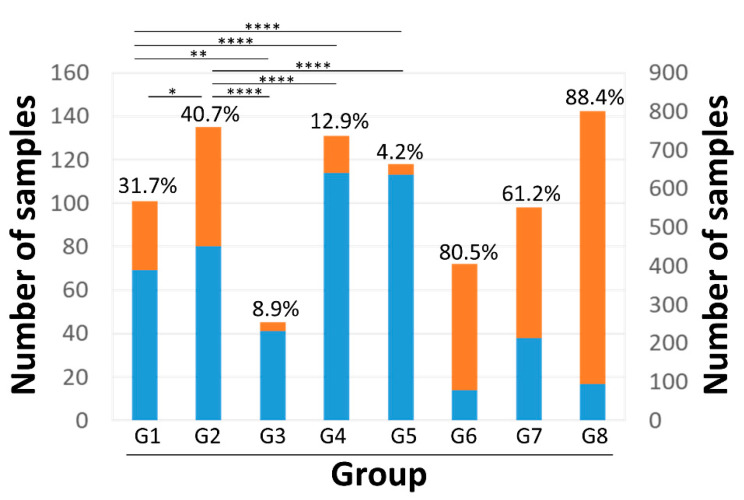
Prevalence of TTMV/TTMDV-expanded anellomes among different patient groups. The proportion of samples with (orange) and without (blue) TTMV/TTMDV-expanded anellomes was presented for each group. Asterisk indicated the *p* values of the between-group comparisons (*, *p* < 0.5; **, *p* < 0.1; ****, *p* < 0.001). The group G8 is represented using the right y-axis. Groups G1–8 stand for acute liver failure, liver transplantation, kidney transplantation, lung transplantation, blood donors, and blood donors from Spain, dengue-like symptoms, and febrile children, respectively.

We found that 160 (10.7%), 246 (16.4%), and 1095 (72.9%) of the 1501 NGS data that we analyzed contained one, two, and three AV genera, respectively. The relative abundance of combinational TTMV and TTMDV within an anellome differed significantly between the patient groups (*p* = 6.99 × 10^−124^, Kruskal–Wallis test). Apparent skewness was also observed within patient groups (Figure S3). Therefore, the data distribution patterns were explored in 530 NGS data (G1–5) from adult subjects without the use of pooling samples in NGS library preparation (Table 2). The histogram of relative abundance of TTMV and TTMDV exhibited an exponential distribution (*p* = 0.372 using Kolmogorov–Smirnov test, indicating that the hypothesis cannot be rejected) (Figure 5). The lower bound point (χ_min_) was calculated to be 0.32 (32%) under the continuous exponential distribution model (Figure 5). Using this value as a cutoff to define a TTMV/TTMDV-expanded anellome, we found the prevalence of the TTMV/TTMDV-expanded anellome to be significantly higher among two liver cohorts compared with other groups: 31.7% and 40.7% in G1 and G2, respectively (Figure 6). The highest rates of the TTMV/TTMDV-expanded anellome were observed in the G6, G7, and G8 groups (80.5%, 61.2%, and 88.4%, respectively) (Figure 6). However, it should be noted that both G6 and G7 used pooled samples for NGS library preparation. Group G8 was febrile children from Africa (Table 2).

### 3.4. Longitudinal Anellome Evolutionary Patterns in Patients Undergoing Liver Transplantation

**Figure 7:**
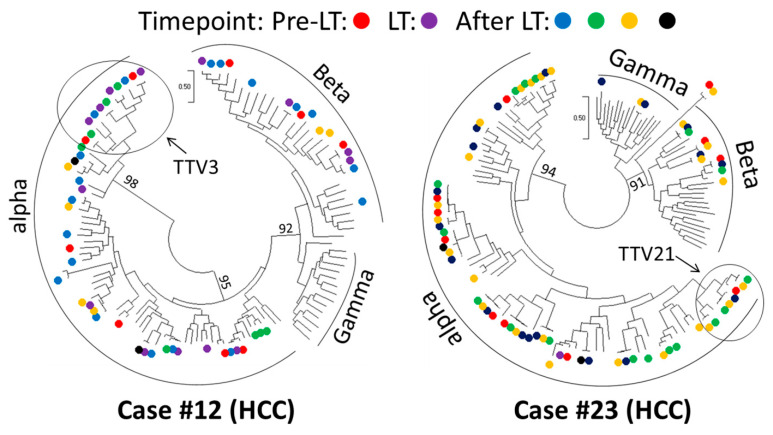
Phylogenetic representation of anellovirus evolutionary dynamics in liver transplantation. Trees were constructed using anellovirus ORF1 sequences of two selected cases (#12 and #23). Time points are indicated using different colors; unlabeled branches show anellovirus reference sequences. AV sequences belonging to the same AV species, exemplified using Torque teno virus 3 (TTV3) in case #12 and Torque teno virus 21 (TTV21) in case #23, were detected at multiple time points before and after liver transplantation. Bootstrap values are indicated on major branches.

In the liver transplantation cohort, we selected two patients (cases #12 and #23) in which the AV ORF1 was detected at all six time points before and after surgery. At 90% nucleotide similarity, a total of 57 and 76 AV ORF1 sequences were harvested from cases #12 and #23, respectively. These sequences covered 18 AV species in each case. Phylogenetic trees revealed AV OFR1 sequences from different time points to be clustered together, supporting a spatial pattern (Figure 7). As exemplified using TTV3 in case #12 and TTV21 in case #23, AV sequences belonging to the same species were detected at multiple time points before and after liver transplantation (Figure 7).

## 4. Discussion

We have recently developed improved methods of AV detection and analysis that overcome the technical challenges associated with the exceptional genome diversity of this family of viruses [6]. These methods enabled us to examine the anellomes in 12 paired serum/liver samples, as well as in 1646 NGS datasets extracted from 10 serum/plasma metagenomics studies. Given the increased Shannon entropy values, liver anellomes were more diverse than those in serum anellomes, although this difference was not statistically significant. Consistent with previous reports [36], we found the AV titer to be significantly higher in liver than serum samples. In contrast to non-hepatotropic viruses such as hepatitis G virus, hepatotropic viruses like HCV usually have a higher titer in tissue than that in serum [37]. Therefore, our data support the assumption that the liver is one of replicative sites for AV, which appears to have broad tissue tropism [2]. Furthermore, the comparable richness and minimal beta diversity of serum and liver anellomes suggest that the serum anellome composition is representative of that of the liver anellome, at least at the level of AV species. Serum sampling may, therefore, be a less invasive way of evaluating the liver anellome.

We found anellomes to be highly individualized, and co-infection with multiple AV species and genera was common. Consequently, considerable heterogeneity exists among and between patient groups in terms of anellome alpha and beta diversity. An important finding was the increased prevalence of the TTMV/TTMDV-expanded anellome among liver patients, and much higher rates of this anellome among blood donors from Spain (G6), patients with dengue-like symptoms from Brazil (G7), and febrile children from Africa (G8). For groups G6 and G7, approximately four to ten samples were pooled in NGS library preparation [29,30,31]. Group G8 were all children from Africa [35]. It is unclear if the relative abundance of TTMV and TTMDV within an anellome is affected by a pooling strategy in NGS library construction or whether it is an authentic reflection of geographic, ethnic, or age-related differences. Composition of the gut microbiome is shaped by multiple factors including genetics, lifestyle, and the environment [38]; therefore, it is conceivable that the anellome, as its viral counterpart, may be similarly affected [39].

We previously demonstrated the dominance of the TTMV/TTMDV-expanded anellome in the context of HCV-associated HCC [6]. In contrast, among HCV patients without cirrhosis or HCC, this anellome was only observed in 1 of 22 patients (4.5%) from current and previous studies. Taken together, the TTMV/TTMDV-expanded anellome seems to be enriched in liver disease with severe clinical outcomes, although the causality of this enrichment is unclear. AV strains have been reported to persist in longitudinally collected samples [40]. We observed a spatial evolutionary pattern in samples from two patients who received liver transplantation. Acute liver failure has an acute disease onset that may not entail enough time to allow for productive anellome adaption. We therefore speculate that the TTMV/TTMDV-expanded anellome may be a modulator of disease outcome rather than an adaption. Phylogenetically, TTMV and TTMDV are clustered together, separated from the TTV [6]. Given their remarkable genetic distances, AV genera may have considerably different biological properties; in vitro experiments support this, having shown apoptosis to be induced by species from TTV, but not by those from TTMV or TTMDV [41,42]. Thus, anellome composition may be an important factor in liver disease progression.

The present study has some limitations that should be acknowledged. We used bulk tissue samples, which do not enable resolution at the single-cell level. The 10 serum/plasma metagenomics studies identified in the literature search used different experimental protocols in terms of sample processing, amplification method, and NGS library preparation strategy. Random PCR was applied in G1, G2, and G7, while MDA or RCA was used for G3, G4, G5, and G6. The TruePrime WGA kit, utilized in G6, is essentially a type of MDA in which primers are intrinsically generated through the use of a DNA primase [43]. G8 had no amplification step prior to library preparation. While there is no special link between amplification methods and the rate of TTMV/TTMDV-expanded anellome among the eight groups, their subtle influence on the NGS data and therefore our analysis cannot be completely excluded. For instance, in blood donors from the USA that were assigned into G5, Arze et al. used 12 AV-derived primers from the entire genomes to enrich AV sequences [31]. The use of multiple AV primers from highly diversified genomes could result in amplification bias. Taken together, these studies included in our analysis may have differential efficiency for the recovery of AV sequences from patient serum or plasma samples. These variances may introduce potential biases that affect our analysis of anellome composition. Therefore, our observations require further validation in future studies under more appropriate experimental designs to control potential confounding factors, such as age, sex, geographics, ethnics, and methods for sample preparation and sequencing pipelines.

The viral reservoir of AV in humans is estimated to be around 10^10^ viruses with a daily turnover rate of 90% [44]. Yet, the role of the virus in human disease and health is unknown. Currently, AV titers are considered an indicator of immune competence, mainly in the setting of solid organ transplantation [45]. We present insights into the human anellome, which may represent a biological variable linked to clinical outcomes of certain diseases, such as liver disease. The consistency of the anellome composition between serum and liver samples indicates that liquid biopsy would enable longitudinal investigations and increase our knowledge of this mysterious virus in liver disease and other pathologies.

## Figures and Tables

**Table 1:** Summary of clinical demographics and viral readouts in 12 patients with chronic HCV infection.

Pt.	Age	Sex	Liver Histology *		HCV		Anellovirus					
							Richness **		Shannon Index **		Titer	
			Stage	Grade	Gen.	Titer (×10^5^ Copies/mL)	Liver	Serum	Liver	Serum	Liver (×10^5^ Copies/g)	Serum (×10^5^ Copies/mL)
1	51	M	2	2	1a	4.41	28	29	0.66	0.69	15.63	3.13
2	50	M	2	2	1a	12.44	30	30	0.67	0.71	0.44	0.04
3	57	M	2	4	1a	5.42	36	35	0.8	0.79	1.05	0.31
4	49	M	2	2	1a	29.80	38	38	0.61	0.61	0.99	0.2
5	62	F	2	1	1a	1.86	2	1	1	0	2.77	0.28
6	56	F	2	2	1a	0.35	27	24	0.53	0.55	10.48	0.53
7	25	M	2	1	1a	11.26	7	7	0.48	0.29	1.81	0.36
8	32	F	2	1	1a	3.25	7	4	0.57	0.35	5.44	0.54
9	61	F	2	0	1a	25.83	5	2	0.58	0.28	3.95	0.31
10	54	F	3	3	1a	1.73	17	21	0.58	0.47	1.26	0.29
11	54	M	3	4	1a	9.59	22	23	0.52	0.47	1.64	0.33
12	43	F	1	1	1a	3.76	14	17	0.57	0.58	6.51	0.72

* Liver histology from biopsy tissue was evaluated according to Scheuer’s scoring system. ** The richness and Shannon entropy were summarized at the level of AV species. Pt, patient; Gen., genotype.

**Table 2:** Summary of ten serum or plasma metagenomics studies. PCR, polymerase chain reaction; RCA, rolling circle amplification; MDA, multiple displacement amplification; WGA, whole genome amplification; AV, anellovirus; Ref, reference.

Group	SRA#	Clinical Diagnosis	Sample					NGS Library		Ref.
			Place	Number	Type	Pretreatment	Amplification	Number	Pooled	
G1	PRJNA389455	Acute liver failure	USA	150	plasma	DNase	Random PCR	150	No	[26]
G2	PRJNA660895	liver transplantation	Belgium	24	plasma	Nucleases	Random PCR	140	No	[23]
G3	PRJNA605928	kidney transplantation	France	30	plasma	DNases/RNases	RCA	64	No	[27]
G4	PRJNA390659 PRJNA419524	lung transplantation	USA	50	serum	No	RCA	155	No	[28,29]
G5	PRJNA526976	blood donors	China	90	serum	No	MDA	90	No	[30]
	PRJNA679286	blood donors	USA	53	serum	No	AV-RCA	57	No	[31]
G6	PRJNA691135 PRJNA731624	blood donors	Spain	707	plasma	No	TruePrime WGA	72	Yes	[32,33]
G7	PRJNA602336	dengue-like symptom	Brazil	781	plasma	DNases/RNases	Random PCR	102	Yes	[34]
G8	PRJNA666535	febrile children	Tanzania	816	serum	DNase	No	816	No	[35]

## Data Availability

Raw sequence data after quality control and the removal of human sequences were deposited in fastq format in the NCBI Sequence Read Archive (SRA) under BioProject ID: PRJNA749275.

## Supplementary Materials

The following supporting information can be downloaded at: https://www.mdpi.com/article/10.3390/v15081635/s1, Figure S1. Pairwise comparison of Bray–Curtis (A) or Yue–Clayton (B) dissimilarity among ten groups, including paired serum/liver samples; Figure S2. Frequency and relative abundance (average) of 79 AV reference species among 1501 NGS data; Figure S3. Box and whisker plots of the relative abundance of combinational TTMV and TTMDV among patient groups. Dots indicate outliers. Among-group comparison was carried out with Kruskal–Wallis test; Table S1. Summary of pairwise comparison of anellome richness and Shannon entropy via Wilcoxon test among eight groups; scripts for the computation of anellome beta diversity.
