# Supplementary material for: Expansion of Betatorquevirus and/or Gammatorquevirus in Patients with Severe Clinical Outcomes of the Liver Diseases
Source: Viruses. 2023 Jul 27;15(8):1635. doi: 10.3390/v15081635 (PMC10457780; doi:10.3390/v15081635)
Supplement: Supplementary file 1 [file viruses-15-01635-s001.zip › viruses-2452249-supplementary.pdf]

Title: Expansion of *Betatorquevirus and/or Gammatorquevirus* in Patients with Severe Clinical Outcomes of the Liver Diseases

Supporting information

Table S1. Summary of pairwise comparison of anellome richness and Shannon entropy via Wilcoxon test among eight groups.

Figure S1. Pairwise comparison of Bray-Curtiss (A) or Yue-Clayton (B) dissimilarity among ten groups, including paired serum/liver samples.

Figure S2. Frequency and relative abundance (average) of 79 AV reference species among 1501 NGS data.

Figure S3. Box and whisker plots of the relative abundance of combinational TTMV and TTMDV among clinical phenotypes. Dots indicate outliers. Among-group comparison was done with Kruskal-Wallis test.

Scripts for the computation of anellome beta diversity

No	group1	group2	n1	n2	Number of AV species (richness)				Shannon entropy			
					statistic	p	p.adj	p.adj.signif	statistic	p	p.adj	p.adj.signif
1	G1	G2	101	135	2950.5	8.00E-14	2.24E-12	****	4459	5.48E-06	0.000153	***
2	G1	G3	101	45	1891.5	0.103	1	ns	2555	0.231	1	ns
3	G1	G4	101	131	2919	2.61E-13	7.31E-12	****	5187	0.005	0.135	ns
4	G1	G5	101	118	2415.5	2.79E-14	7.81E-13	****	3939.5	1.55E-05	0.000434	***
5	G1	G6	101	72	880.5	1.73E-17	4.84E-16	****	1903	9.35E-08	2.62E-06	****
6	G1	G7	101	98	1893.5	4.57E-14	1.28E-12	****	3099	5.22E-06	0.000146	***
7	G1	G8	101	801	2858	1.98E-52	5.54E-51	****	5190.5	2.51E-46	7.03E-45	****
8	G2	G3	135	45	4576.5	3.58E-07	1.00E-05	****	4567	4.38E-07	1.23E-05	****
9	G2	G4	135	131	9143.5	0.632	1	ns	10243	0.026	0.717	ns
10	G2	G5	135	118	7051	0.115	1	ns	8102.5	0.813	1	ns
11	G2	G6	135	72	3277	0.000114	0.003	**	3937	0.025	0.689	ns
12	G2	G7	135	98	6740.5	0.805	1	ns	6498	0.819	1	ns
13	G2	G8	135	801	11242	3.59E-49	1.01E-47	****	11689	3.55E-48	9.94E-47	****
14	G3	G4	45	131	1443.5	3.29E-07	9.21E-06	****	1859	0.000224	0.006	**
15	G3	G5	45	118	1221	9.79E-08	2.74E-06	****	1333	9.30E-07	2.60E-05	****
16	G3	G6	45	72	412.5	1.28E-11	3.58E-10	****	601	1.15E-08	3.22E-07	****
17	G3	G7	45	98	939	3.59E-08	1.01E-06	****	997	1.53E-07	4.28E-06	****
18	G3	G8	45	801	975.5	1.15E-26	3.22E-25	****	883	6.25E-27	1.75E-25	****
19	G4	G5	131	118	6563	0.04	1	ns	6635	0.054	1	ns
20	G4	G6	131	72	2986	1.54E-05	0.000431	***	3117	6.55E-05	0.002	**
21	G4	G7	131	98	6405	0.978	1	ns	5265	0.02	0.56	ns
22	G4	G8	131	801	8859.5	1.24E-52	3.47E-51	****	7321	2.87E-56	8.04E-55	****
23	G5	G6	118	72	3497	0.041	1	ns	3297	0.01	0.273	ns
24	G5	G7	118	98	6554.5	0.091	1	ns	5606	0.701	1	ns
25	G5	G8	118	801	15482	3.63E-32	1.02E-30	****	8923.5	5.11E-46	1.43E-44	****
26	G6	G7	72	98	4855.5	2.81E-05	0.000787	***	4148	0.051	1	ns
27	G6	G8	72	801	11211.5	7.95E-18	2.23E-16	****	9947	3.08E-20	8.62E-19	****
28	G7	G8	98	801	6808.5	8.89E-41	2.49E-39	****	8675	2.10E-36	5.88E-35	****

Table S1. Summary of pairwise comparison of anellome richness and Shannon entropy via Wilcoxon test among eight groups. ns, not significant.

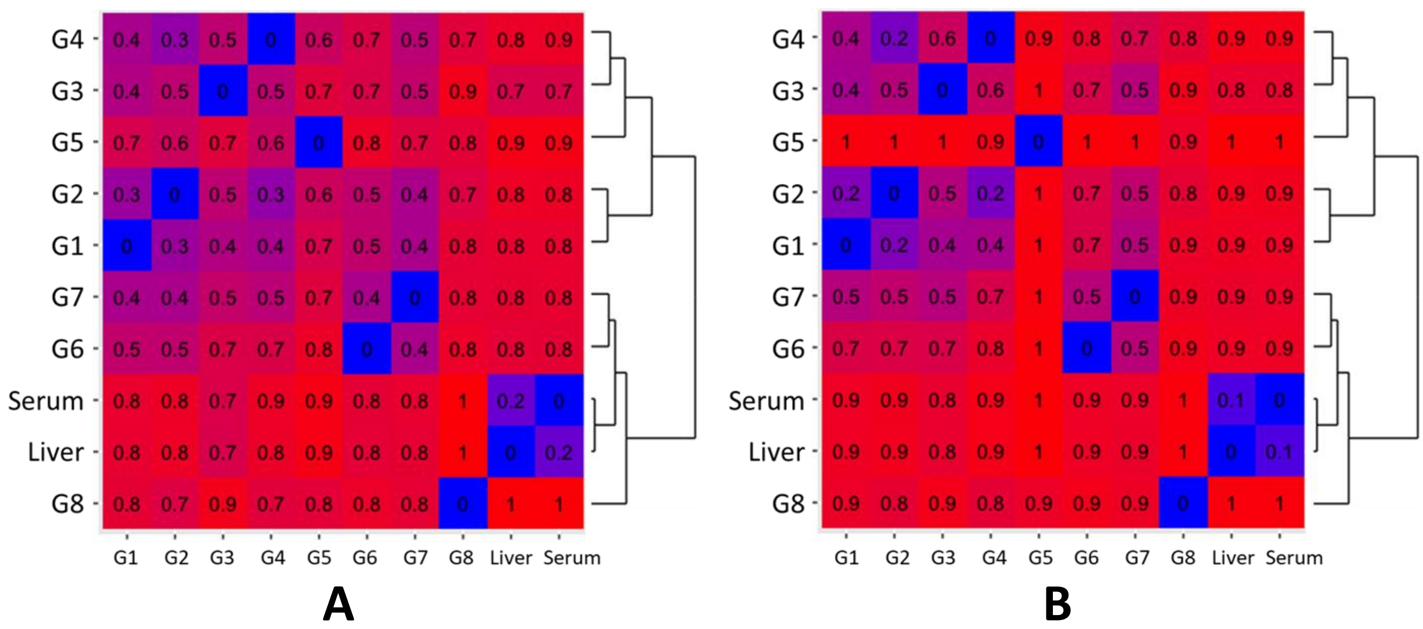

Figure S1. Pairwise comparison of Bray-Curtiss (A) or Yue-Clayton (B) dissimilarity among ten groups, including paired serum/liver samples. The dissimilarity was represented by the heatmap with hierarchical clustering of all groups.

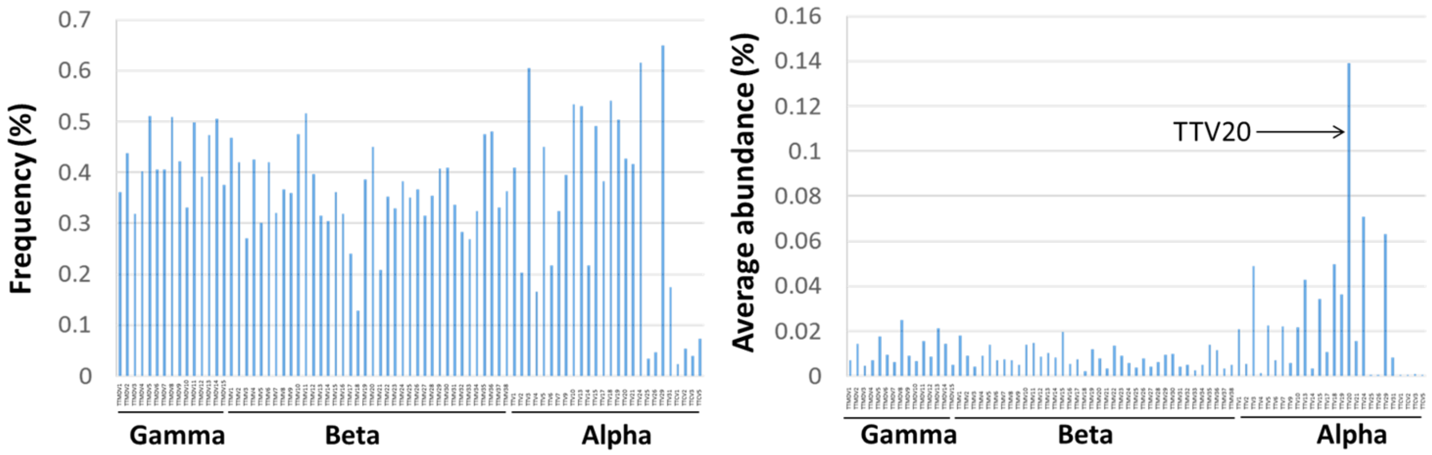

Figure S2. Frequency (left) and relative abundance (average) (right) of 79 AV reference species among 1,501 NGS data

Kruskal-Wallis test p=6.99x10^{-124}

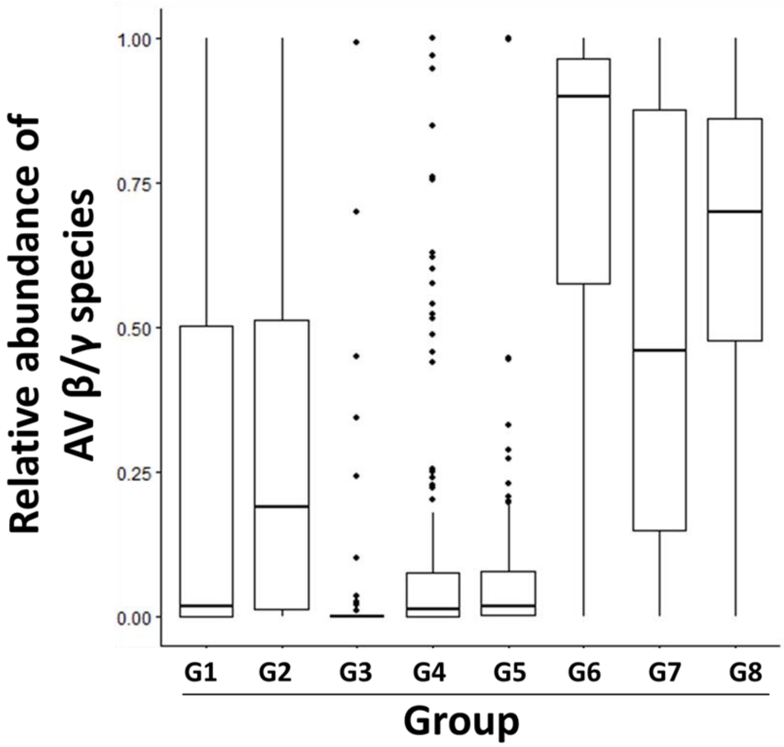

Figure S3. Box and whisker plots of the relative abundance of combinational AV *Betatorquevirus* and *Gammatorquevirus* genera among patient groups. Dots indicate outliers. Among-group comparison was done with Kruskal-Wallis test.

Scripts for the computation of anellome beta diversity

```
#!/usr/local/bin/python3

import numpy as np
import pandas as pd

#first row: 79 AV species
#first column: classified into 9 groups (1501 subjects)
#each row == one subject
#calculate beta diversity and make between-group comparisons (maybe in heatmap)

#beta-diversity calc: bray curtis parameter

#Main Goal: id new virus, trying to demonstrate whether this virus increases liver disease likelihood

#questions: what does each value represent? Is instance more important than actual value quantity?
#any value in comparing diversity within group? probably between group right?
#can each group be considered an environment with the top row being the different populations?

#A:
#subspecies 1-n, sort of like batting percentage, number of viruses seen for every virus checked (reads) analog virus or TTV
```

```
#check if each row adds to 1
#each group based on observed phenotypes pertaining liver, pathology observed in liver

#measure both inter/intragroup betas, make histogram of number of unique virus strains in group (alpha diversity)
#vertical series of dots, calc mean and std for groups 1-9
#make heatmap of grid, should be traingle, same number should b white, should be 9C2

#calculate beta diversity for each group + mean and std (1-9)
#then intergroup comparison with heatmap
#compare liver vs. liver+serum for detection purposes

def alpha():
    data = pd.read_excel("~/Desktop/aurora lab/Sum_composition1501.xlsx")
    #transpose to scale xlsx through columns, not rows
    data_t = data.transpose()

    #makes list of list of [group, alpha-div]
    alpha_list = []

    #go through each col and count alpha-div, make list and append to list
    for i in range(len(data)):
        col = data_t[i]
        count = col[col > 0].count() - 1

        alpha_list.append([data_t[i].iat[0], count])

    #convert list of list to dataframe, then output to xlsx
    alpha_div = pd.DataFrame(alpha_list, columns = ["Group", "alpha-div"])
    alpha_div.index += 1
    alpha_div.to_excel("~/Desktop/aurora lab/alpha_liver.xlsx")




def beta():
    data = pd.read_excel("~/Desktop/aurora lab/Sum_composition1501.xlsx")
    data_t = data.transpose()
    #print(data_t)

    #makes empty list to store beta values
    bcbeta_list = []
    ycbeta_list = []

    group1 = [0]*80
    group2 = [0]*80
    group3 = [0]*80
    group4 = [0]*80
    group5 = [0]*80
    group6 = [0]*80
    group7 = [0]*80
    group8 = [0]*80
    groupL = [0]*80
    groupS = [0]*80
```

```
#sorts data into a list for each group
for i in range(len(data)):
    val_list = data_t[i].tolist()
    for j in range(len(val_list)):
        if val_list[0] == 1:
            group1[j] += val_list[j]
        elif val_list[0] == 2:
            group2[j] += val_list[j]
        elif val_list[0] == 3:
            group3[j] += val_list[j]
        elif val_list[0] == 4:
            group4[j] += val_list[j]
        elif val_list[0] == 5:
            group5[j] += val_list[j]
        elif val_list[0] == 6:
            group6[j] += val_list[j]
        elif val_list[0] == 7:
            group7[j] += val_list[j]
        elif val_list[0] == 8:
            group8[j] += val_list[j]
        elif val_list[0] == "Liver":
            if val_list[j] != "Liver":
                groupL[j] += val_list[j]
        elif val_list[0] == "Serum":
            if val_list[j] != "Serum":
                groupS[j] += val_list[j]
        else:
            print("something went wrong")

#9C2 = 36 possible combinations
#1/2 1/3 1/4 1/5 1/6 1/7 1/8 1/9 (8)
#2/3 2/4 2/5 2/6 2/7 2/8 2/9 (7)
#3/4 3/5 3/6 3/7 3/8 3/9 (6)
#4/5 4/6 4/7 4/8 4/9 (5)
#5/6 5/7 5/8 5/9 (4)
#6/7 6/8 6/9 (3)
#7/8 7/9 (2)
#8/9 (1)

#removes error sum at index 0
group1.pop(0)
group2.pop(0)
group3.pop(0)
group4.pop(0)
group5.pop(0)
group6.pop(0)
group7.pop(0)
group8.pop(0)
groupL.pop(0)
groupS.pop(0)
```

```
    a = [group1, group2, group3, group4, group5, group6, group7, group8, groupL, groupS]
    b = [group1, group2, group3, group4, group5, group6, group7, group8, groupL, groupS]

    #2C9 and calculates BC beta-diversity
    for i in range(1,11):
        for j in range(1,11):
            bcbeta_list.append(bc(i, j, a, b))

    #2C9 and calculates YC beta-diversity
    for i in range(1,11):
        for j in range(1,11):
            ycbeta_list.append(yc(i, j, a, b))

    #creates xlsx of the sum of each strain per group
    groups = []
    for i in range(len(group1)):
        groups.append([group1[i], group2[i], group3[i], group4[i], group5[i], group6[i], group7[i], group8[i], groupL[i],
groupS[i]])
    groups = pd.DataFrame(groups, columns = ["Group 1", "Group 2", "Group 3", "Group 4", "Group 5", "Group 6", "Group
7", "Group 8", "Liver", "Serum"])
    groups.index += 1
    groups.to_excel("~/Desktop/aurora lab/groups.xlsx")

    #creates xlsx of groups compared and calculated BC beta-div
    bcbeta_div = pd.DataFrame(bcbeta_list, columns = ["Group A", "Group B", "beta-div"])
    bcbeta_div.index += 1
    bcbeta_div.to_excel("~/Desktop/aurora lab/bc_beta_liver.xlsx")

    #creates xlsx of groups compared and calculated YC beta-div
    ycbeta_div = pd.DataFrame(ycbeta_list, columns = ["Group A", "Group B", "beta-div"])
    ycbeta_div.index += 1
    ycbeta_div.to_excel("~/Desktop/aurora lab/yc_beta_liver.xlsx")


def bc(i, j, a, b):
    num_sum = 0
    den_sum = 0

    beta = 0

    for count, val in enumerate(a[i-1]):
        num_sum += min(val, b[j-1][count])

    den_sum = sum(a[i-1]) + sum(b[j-1])

    beta = 1 - 2 * (num_sum / den_sum)

    if i == 9:
        i = "Liver"
    elif i == 10:
        i = "Serum"
```

```
    if j == 9:
        j = "Liver"
    elif j == 10:
        j = "Serum"

    vals = [i, j, beta]
    return vals

def yc(i, j, a, b):
    num_sum = 0
    den_sum = 0

    xy_sum = 0
    xy2_sum = 0

    beta = 0

    for count, val in enumerate(a[i-1]):
        xy_sum += val * b[j-1][count]
        xy2_sum += (val - b[j-1][count]) * (val - b[j-1][count])

    num_sum = xy_sum
    den_sum = xy_sum + xy2_sum

    beta = 1 - num_sum / den_sum

    if i == 9:
        i = "Liver"
    if i == 10:
        i = "Serum"

    if j == 9:
        j = "Liver"
    if j == 10:
        j = "Serum"

    vals = [i, j, beta]
    return vals



#alpha()
beta()
```
